# Acquired reactive perforating collagenosis in association with prostate adenocarcinoma, chronic lymphocytic leukemia, and Graves’ disease^☆^^☆☆^

**DOI:** 10.1016/j.abd.2019.09.029

**Published:** 2020-04-18

**Authors:** Leyla Huseynova, Neslihan Akdogan, Özay Gököz, Sibel Ersoy Evans

**Affiliations:** a Department of Dermatology and Venerology, Faculty of Medicine, Hacettepe University, Ankara, Turkey; b Department of Pathology, Faculty of Medicine, Hacettepe University, Ankara, Turkey

**Keywords:** Graves disease, Leukemia lymphocytic chronic b-cell, Neoplasms/secondary, Prostatic neoplasms, Pruritus

## Abstract

Acquired reactive perforating collagenosis is a rare skin disorder characterized by the presence of umbilicated pruritic papules and nodules. Transepidermal elimination of altered and perforating bundles of basophilic collagen from the epidermis is a characteristic histologic feature of acquired reactive perforating collagenosis. Along with its well-known association with systemic diseases such as diabetes mellitus, chronic renal failure, and dermatomyositis, there are reports of acquired reactive perforating collagenosis being associated with malignancies. Herein, we present a case of acquired reactive perforating collagenosis associated with chronic lymphocytic leukemia, prostate adenocarcinoma, and Graves's disease. Clinicians are required to be more vigilant in evaluating patients with acquired reactive perforating collagenosis due to its unique association with malignancies and other systemic diseases.

## Introduction

Acquired reactive perforating collagenosis (ARPC) is a rare skin disorder with characteristic clinical and histological features and a typical adult-onset. ARPC is one of the four diseases that belong to the disease group called primary perforating dermatosis. Patients commonly present with characteristic umbilicated necrotic papules or nodules having a localized or generalized distribution. Histopathological examination of these lesions exhibits transepidermal elimination of altered and perforating bundles of basophilic collagen from the epidermis.^1^ Although the etiology and pathogenesis of ARPC are not fully understood, the disease is commonly associated with systemic diseases such as diabetes mellitus (DM), chronic renal insufficiency, dermatomyositis, hyperuricemia, and malignancies.

## Case report

A 75-year-old male was admitted to our dermatology department with a widespread, itchy rash that had developed during five months. The patient did not respond well to oral antihistamine, topical corticosteroid cream, and narrowband ultraviolet B phototherapy (UVB) for 13 sessions. He was diagnosed with chronic lymphocytic leukemia (CLL) and Graves’ disease (GD) 11 and 12 years ago, respectively. He was on drug-free follow-up for CLL and GD at the time of hospital admission. The patient was otherwise healthy with no family history of diseases.

**Figure 1:**
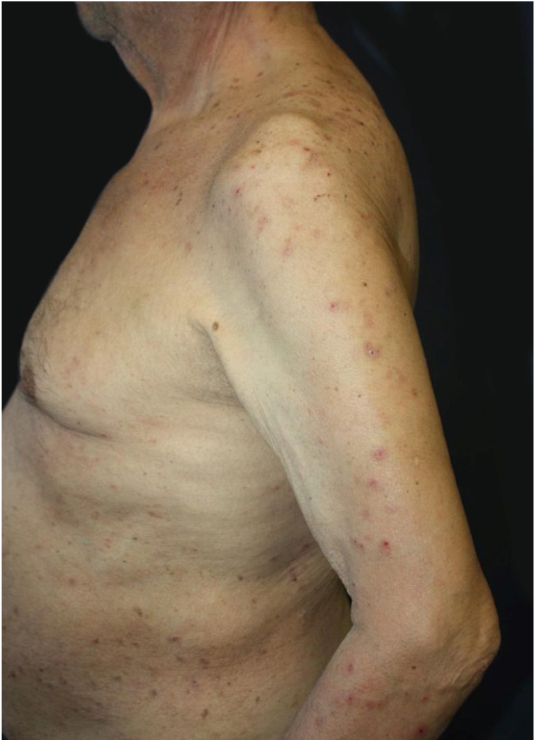
Widespread excoriations and dome-shaped papules with central crusts on the trunk and upper extremity.

**Figure 2:**
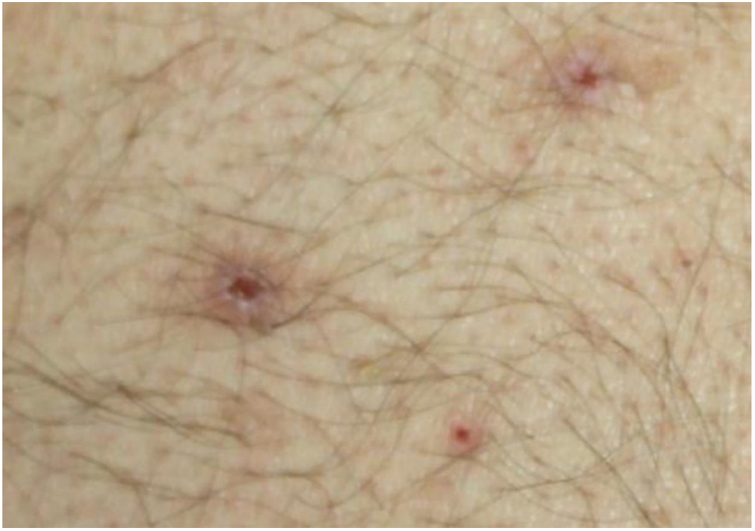
Umbilicated papules with central hemorrhagic crust.

**Figure 3:**
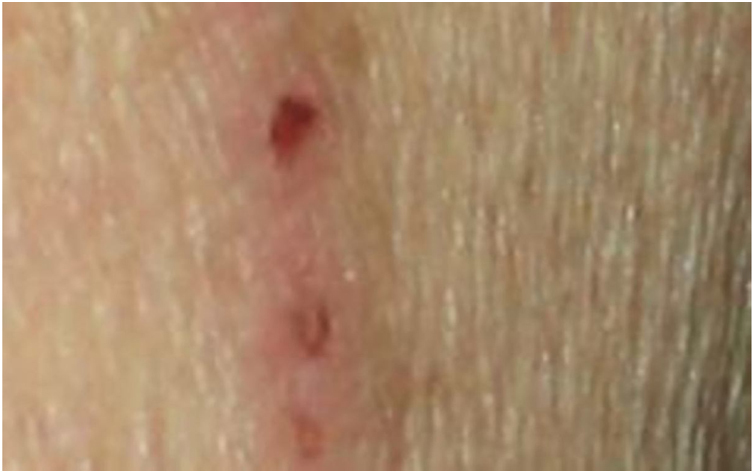
Linear distribution of some papules.

Dermatological examination revealed multiple, widespread, dome-shaped umbilicated papules and nodules with an average diameter of ∼4 mm distributed on the trunk, face, and upper and lower extremities (Figure 1, Figure 2). Some of these were located in a linear pattern suggesting the Koebner phenomenon (Fig. 3). Examination of the nails and oral mucosa revealed no abnormalities.

**Figure 4:**
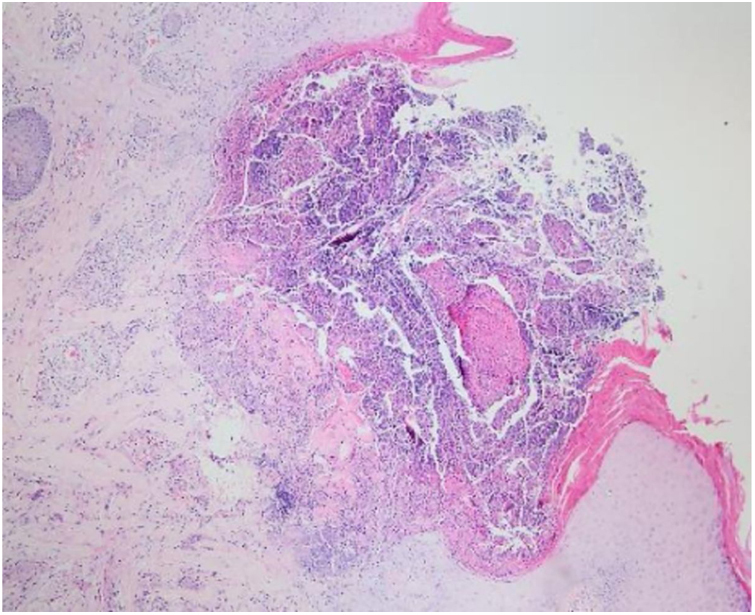
Punched-out ulceration with cellular debris in the crateriform invagination (Hematoxylin & eosin, ×40).

**Figure 5:**
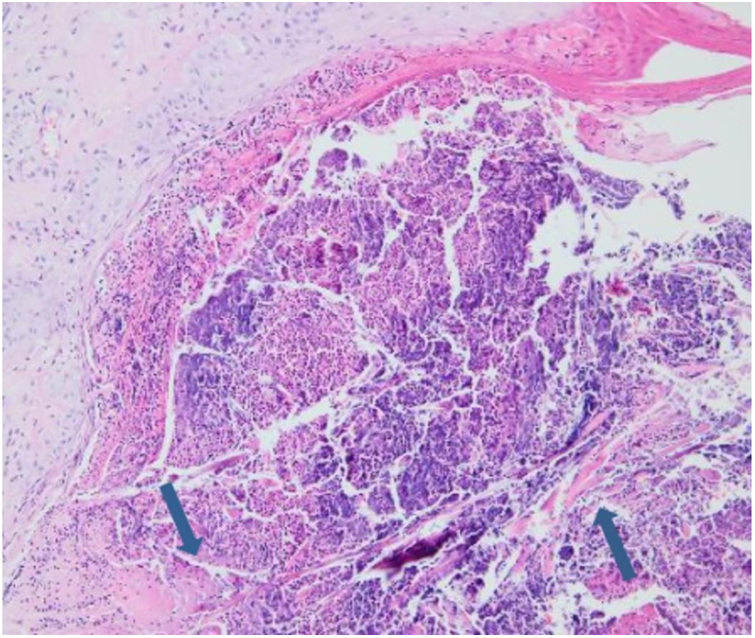
Collagen fibers traversing from the dermis through the ulcer to the surface (Hematoxylin & eosin, ×200).

Laboratory results of complete hemogram, blood sugar, liver, and kidney function tests were found to be within normal limits. Histopathological evaluation of a representative skin lesion taken from the left leg showed acanthosis, basket weave orthokeratosis, increased number of vessels in the superficial dermis, and perivascular and interstitial inflammatory infiltrate with erythrocyte extravasation. Punched-out ulceration with cellular debris was observed in the crateriform invagination and transepidermal elimination of collagen fibers (Figure 4, Figure 5). ARPC was diagnosed based on the clinical and histological findings and the onset of the disease at the age of 75 years. Treatment with oral acitretin 25 mg/day was planned; however, the patient was lost to follow-up. Later, during a control visit to our clinic, an excellent response to gabapentin 300 mg/day and topical doxepin cream for one month was observed in the patient; these medications were prescribed in another medical center. Meanwhile, a diagnosis of prostate adenocarcinoma was confirmed following prostate biopsy due to elevated serum levels of prostate-specific antigen.

## Discussion

Reactive perforating collagenosis (RPC) can be both hereditary (familial) and acquired.^1^, ^2^ Hereditary RPC is a rare form that is inherited in an autosomal recessive pattern and occurs in infancy or early childhood. ARPC is more common than the hereditary form; unlike the hereditary form, ARPC is characterized by a typical adult-onset. The prevalence and incidence of ARPC are unknown; sex distribution is thought to be equal.

Transepidermal elimination of altered dermal collagen is thought to be crucial for the pathogenesis of ARPC. However, the etiology and pathogenesis of ARPC are not fully understood. Tsuboi et al. suggested that systemic diseases contribute to the degeneration of collagen fibers and the production of altered dermal products either directly or indirectly.^3^ Some authors suggest microtrauma caused by pruritus could trigger ARPC in predisposed patients.^4^ Improvement of lesions after antipruritic treatment supports this theory. Microangiopathy due to DM is thought to be other cause of ARPC as high glucose levels increase glycosylation of proteins and other compounds resulting in hyalinization and alterations (cross-linking) in collagen structure.^5^ Other proposed mechanisms for the pathogenesis of ARPC include overexpression of transforming growth factor beta 3, dermal and epidermal changes related to metabolic disorders, and the dermal micro-deposits, such as calcium, that are not removed during dialysis in patients with renal failure.^5^

ARPC is commonly associated with systemic diseases such as DM, chronic renal failure, and dermatomyositis. Review of the literature revealed 14 published cases of ARPC that were associated with malignancies. Despite the small number of these cases, some authors suggest to consider ARPC as a paraneoplastic syndrome.^6^ Hodgkin's lymphoma is reported to be the most common malignancy associated with ARPC; in addition, leukemia, periampullary carcinoma, thyroid carcinoma, colon carcinoma, hepatocellular carcinoma, prostate carcinoma, and adenocarcinoma of unknown primary site have also been reported in patients with ARPC.^6^, ^7^, ^8^, ^9^ It is unknown whether malignancy itself or pruritus triggered by malignancy leads to the development of ARPC. It is striking that ARPC lesions are preceded by the diagnosis of malignancy in most cases.

In ARPC, lesions are usually widespread and may appear anywhere on the body with a slight predilection for the lower extremities^4^; severe pruritus usually accompanies. New lesions can appear as Koebner's phenomenon due to cutaneous scratching. It is nearly impossible to clinically differentiate ARPC from other forms of perforating dermatosis based on morphology; however, the histologic findings are specific. The differential diagnosis of ARPC consists of disorders presented with umbilicated papules and nodules with central keratotic plaques such as prurigo nodularis, eruptive keratoacanthomas, granuloma annulare, sarcoid, porokeratosis, hyperthrophic lichen planus, Darier's disease, and follicular disorders such as phrynoderma and keratosis pilaris.^1^

A standardized treatment protocol for ARPC does not exist and treatment remains challenging. Controlling pruritus and the underlying extracutaneous disorder is important for the management of ARPC. In drug-induced cases, cessation of the suspected drug is crucial. Topical corticosteroids, oral antihistamines, emollients, UVB phototherapy, systemic retinoids, allopurinol, and cryotherapy are used for the treatment of ARPC.^5^

As far as we know, there is no report on ARPC being associated with CLL and GD; however, a case of ARPC associated with prostate carcinoma has been reported before. Herein, we reported a case of ARPC associated with CLL, prostate adenocarcinoma, and GD mimicking scabies, folliculitis, or prurigo simplex. We emphasize the importance of the association of ARPC with malignancies. Clinicians are required to be more vigilant in evaluating patients with ARPC considering its unique association with malignancies as well as other systemic diseases.

## Financial support

None declared.

## Authors’ contributions

Leyla Huseynova: Approval of the final version of the manuscript; conception and planning of the study; elaboration and writing of the manuscript; obtaining, analysis, and interpretation of the data; effective participation in research orientation; intellectual participation in the propaedeutic and/or therapeutic conduct of the studied cases; critical review of the literature; critical review of the manuscript.

Neslihan Akdogan: Approval of the final version of the manuscript; conception and planning of the study; elaboration and writing of the manuscript; obtaining, analysis, and interpretation of the data; effective participation in research orientation; critical review of the literature; critical review of the manuscript.

Özay Gököz: Approval of the final version of the manuscript; elaboration and writing of the manuscript; obtaining, analysis, and interpretation of the data; effective participation in research orientation; critical review of the literature; critical review of the manuscript.

Sibel Ersoy Evans: Approval of the final version of the manuscript; elaboration and writing of the manuscript; obtaining, analysis, and interpretation of the data; effective participation in research orientation; intellectual participation in the propaedeutic and/or therapeutic conduct of the studied cases; critical review of the literature; critical review of the manuscript.

## Conflicts of interest

None declared.
